# Inorganic Silica Nanoparticles Increase Lysosomal Biology and Protease Activity

**DOI:** 10.3390/ijms26178291

**Published:** 2025-08-26

**Authors:** Anastasiia O. Syrocheva, Valentina I. Gorbacheva, Vera S. Egorova, Andrey A. Zamyatnin, Alessandro Parodi, Ekaterina P. Kolesova

**Affiliations:** 1 Research Center for Translational Medicine, Sirius University of Science and Technology, Sochi 354340, Russia; syrocheva.ao@talantiuspeh.ru (A.O.S.); tinafowl7@gmail.com (V.I.G.); egorova.vs@talantiuspeh.ru (V.S.E.); 2 Faculty of Bioengineering and Bioinformatics, Lomonosov Moscow State University, Moscow 119234, Russia; zamyat@belozersky.msu.ru; 3 Belozersky Institute of Physico-Chemical Biology, Lomonosov Moscow State University, Moscow 119992, Russia; 4 Department of Biochemistry, Sechenov First Moscow State Medical University, Moscow 119991, Russia

**Keywords:** drug delivery, nanocarriers, mesoporous silica nanoparticles, albumin nanoparticles, lysosomes, cathepsins

## Abstract

The use of nanoparticles has revolutionized drug delivery by enabling targeted and controlled therapeutic release. However, their interactions with intracellular organelles, particularly lysosomes, are not yet fully understood. This study delineates the differential effects of two widely used nanocarriers—mesoporous silica (MSNs) and albumin (ANPs) nanoparticles—on lysosomal biology, with a focus on the expression and activity of cathepsins (CtsB and CtsD), which are key proteases involved in protein degradation and maintaining cellular balance. These two types of nanoparticles, differing in their material and degradability, exhibit distinct behaviors inside the cell. We demonstrate that inorganic MSNs cause significant changes in lysosomal function by altering lysosomal content and cathepsin levels, without triggering lysosomal membrane permeabilization—a typical response to organic particle stress. In contrast, ANPs—which are susceptible to lysosomal cathepsin degradation—induce milder changes in cathepsin expression and maintain lysosomal integrity. Our results highlight that the composition of nanocarriers plays a pivotal role in modulating lysosomal protease activity and maintaining overall cellular homeostasis, highlighting the importance of these parameters in the rational design of drug delivery platforms.

## 1. Introduction

The rapid progress of nanomedicine has opened new perspectives for precision therapeutics, with nanoparticle-based drug delivery systems leading this transformation [1,2]. By harnessing the distinctive physicochemical properties of nanostructured materials—such as adjustable size, surface chemistry, and high drug-loading capacity—researchers have developed platforms that overcome many limitations of conventional pharmaceuticals [3,4,5]. These nanocarriers enhance the solubility, stability, and bioavailability of the therapeutic payload, while enabling targeted delivery to specific tissues or cellular compartments, thereby reducing off-target effects and systemic toxicity [6,7]. Both inorganic and organic nanoparticles have demonstrated significant potential in both preclinical and clinical investigations. However, despite these advancements, a comprehensive understanding of the interactions between various nanocarrier compositions and biological systems [8,9], particularly at the subcellular level, remains incomplete. Gaining such insights is crucial for designing next-generation drug delivery systems that maximize therapeutic benefit and safety.

Lysosomes, a cell’s primary degradative organelles [10], play a central role in maintaining metabolic balance [11] through hydrolases and proteolytic enzymes such as cathepsins [12,13]. These proteases not only clear cellular waste but also regulate signaling pathways involved in cell survival, inflammation, and stress responses. As nanomedicine advances, understanding how engineered nanoparticles affect lysosomal function is increasingly important. Among the many nanocarriers studied, silica nanoparticles (MSNs) [14,15] and albumin nanoparticles (ANPs) [16,17] are widely used due to their biocompatibility and tunable properties. Their distinct physicochemical characteristics—rigid, inorganic silica versus protein-based organic albumin—suggest they interact differently with lysosomal biology, particularly in modulating cathepsin expression and activity.

MSNs, known for their mechanical stability [18], have been shown to accumulate within lysosomes, while resisting enzymatic degradation. Their persistence leads to physical overcrowding that disrupts lysosomal ultrastructure and impairs autophagic flux and proteolytic function. Recent studies have shown that MSNs suppress transcription factor EB (TFEB) [19], a key regulator of lysosomal biogenesis, resulting in lysosomal alkalization and the decreased activation of cathepsin B (CtsB) [20]. Additionally, MSN-induced lysosomal membrane permeabilization causes leakage of CtsB into the cytosol, which triggers inflammatory responses via caspase-1 activation, independently of the NLRP3 inflammasome [20]. These findings reveal a paradox: although not biocompatible, MSNs’ physical persistence inside lysosomes initiates a cascade of dysfunction that compromises cellular homeostasis.

In contrast, ANPs have attracted attention as versatile and biocompatible drug delivery vehicles [21]. Their protein-based nature facilitates efficient cellular uptake and enzymatic degradation [22] within lysosomes, primarily through cathepsin activity. This biodegradability supports controlled therapeutic release and reduces the risk of nanoparticle accumulation and toxicity. Furthermore, ANPs interact favorably with intracellular pathways, often modulating lysosomal function without causing structural damage. Our previous work demonstrated that the amount of cross-linker is a key factor determining the enzymatic degradation resistance of nanoparticles [23]. And ANPs increase the expression of cathepsins B, D, and G in endolysosomal compartments, regardless of their degradation kinetics. This biochemical reprogramming may result from ANP-induced redox changes or activation of the mTOR pathway, which influence lysosomal signaling [24]. Importantly, ANPs avoid the physical disruption caused by MSNs, instead producing subtler effects on protease activity that may affect long-term cellular adaptation.

The physicochemical properties of NPs, specifically surface charge, surface area, and hydrophobicity, play a critical role in their interactions with biological systems [4]. Surface charge is particularly important as it influences the interaction with negatively charged cell membranes, thereby affecting cellular internalization and toxicity [25]. A larger surface area—for example, in mesoporous silica nanoparticles due to their porous structure—increases the likelihood and extent of biomolecular interactions [26]. Hydrophobicity governs nanoparticle biocompatibility, stability, and protein adsorption; hydrophobic NPs tend to adsorb more proteins, which may enhance membrane interactions but also elevate toxicity and immunogenic responses [27]. Consequently, surface modification strategies in nanomedicine often aim to increase nanoparticle hydrophilicity to reduce nonspecific protein adsorption and improve biocompatibility [28]. Beyond direct cellular interactions, these physicochemical parameters strongly influence the formation of the protein corona upon exposure to biological fluids, which ultimately determines subsequent bio–nano interactions [29]. The nature of the nanoparticle—whether protein-based or inorganic—significantly affects the corona composition: ANPs, composed of native protein, predominantly recruit homologous proteins such as albumin, forming a stable and biocompatible corona that minimizes nonspecific and enzymatic interactions as well as immune activation [30]. In contrast, MSNs, with their distinct surface chemistry, adsorb a more diverse and dynamic protein repertoire, resulting in a corona characterized by multivalent and nonspecific binding, which facilitates competitive protein interactions. Thus, the nano–bio interface represents a highly complex system governed by multiple interdependent factors.

In this study, we compare the effects of MSNs and ANPs on lysosomal proteolysis and cathepsin expression and activity. Our findings provide a comparative framework to inform the design of nanotherapies that minimize off-target effects. We demonstrate that these two nanoparticle types distinctly influence lysosomal function and enzyme dynamics: inorganic, degradation-resistant MSNs exert pronounced effects on lysosomal biology without causing significant lysosomal destabilization, whereas ANPs induce milder perturbations consistent with their degradability. These divergent lysosomal interactions highlight a critical challenge in nanocarrier design—the need to align material properties with organelle-specific biological responses to optimize therapeutic outcomes.

## 2. Results

### 2.1. Synthesis and Characterization of Nanocarriers (Albumin and Silica NPs)

**Figure 1:**
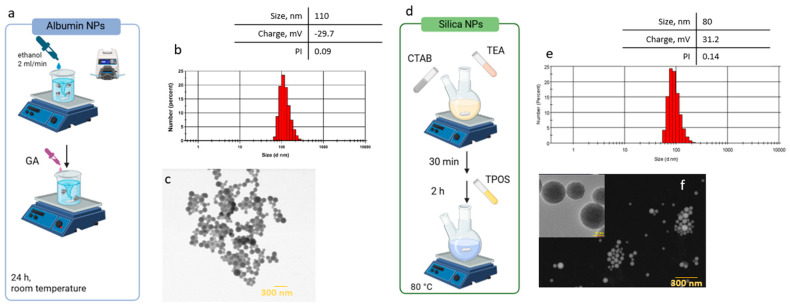
NP synthesis and characterization: (**a**–**c**) ANP synthesis schematic, DLS and Z—potential, and SEM analysis and (**d**–**f**) MSN synthesis schematic, DLS and Z-potential, and SEM analysis; scale bars correspond to 300 nm.

Albumin nanoparticles (ANPs) were synthesized by desolvating bovine serum albumin (BSA) in ethanol and subsequently cross-linking using glutaraldehyde and dispersion into water (Figure 1). Based on our previous studies [23] investigating the effect of cross-linker concentration in ANPs on their interactions with cells, including lysosomal degradation, we selected the minimal concentration that ensures sufficient stability while allowing effective degradation. The particle size was determined using multiple techniques. Both dynamic light scattering and electron microscopy yielded consistent results, showing spherical nanoparticles of approximately 110 nm in diameter (Figure 1). The sample demonstrated high uniformity, as indicated by a low polydispersity index (PDI) of 0.09. This homogeneity likely arises from the strong negative surface charge of the nanoparticles (zeta potential = −29.7 mV), attributed to the predominance of carboxyl groups at pH values above the isoelectric point of BSA (4.7). Such a high surface charge promotes effective electrostatic repulsion between the particles, contributing to excellent colloidal stability.

Mesoporous silica nanoparticles (MSNs) were prepared via the sol–gel method, which typically yields pore sizes in the range of 2 to 5 nm [31]. These particles measured approximately 80 nm with a polydispersity index of 0.14. Surface modification with (APTES) imparted a positive surface charge. Electron microscopy revealed their characteristic porous structure (Figure 1f). Both nanoparticle types exhibit high colloidal stability but differ in surface charge, composition, and degradation behavior within the tumor microenvironment.

### 2.2. Efficiency of Cellular Internalization

**Figure 2:**
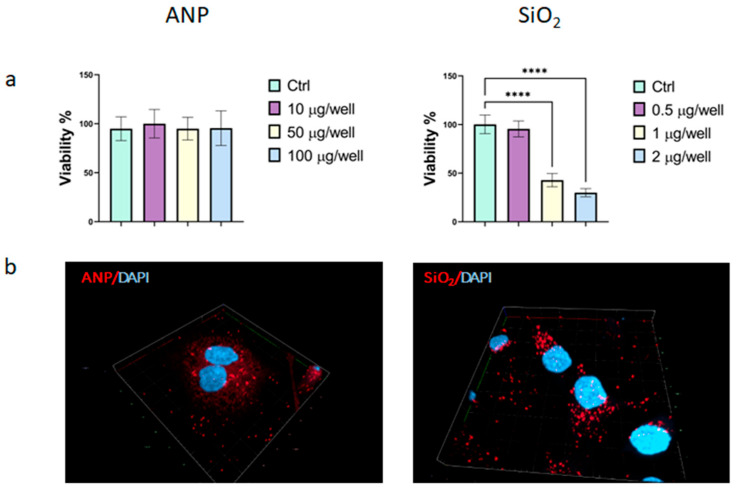
Determination of NPs’ non-toxic doses: (**a**) SKBR3 viability after 72 h incubation with escalating concentrations of ANPs and MSNs (cell density of 10 × 10^3^ cells per well). For each experiment, 3 biological and 6 technical replicates were performed. Results represent mean ± SD. **** = *p* < 0.0001. Significance was calculated via one-way ANOVA, followed by Dunnett’s test. (**b**) Confocal images of SKBR3 cells after 6 h treatment and overnight incubation with Cyanine5.5—labeled NPs (red); after incubation, cells were fixed, and cell nuclei were stained with DAPI (blue). Area is 80 × 80 µm.

To ensure that cytotoxicity would not affect further experiments, we first established the effect of the particles on SKBR3 cell viability. NPs toxicity was evaluated after 72 h of continuous exposure to SKBR3 breast cancer cells. The SKBR-3 cell line serves as an optimal model for investigating the effects of nanoparticles on lysosomal activity and cathepsin expression, owing to its robust endocytic and lysosomal functions. Additionally, its clinical relevance is underscored by inherent resistance to multiple therapies, including chemotherapeutic agents such as paclitaxel and doxorubicin [32,33]. As depicted in Figure 2, the analyzed particles exhibit distinct toxicity profiles. ANPs demonstrated high biocompatibilities, causing no significant changes in cell survival even at 100 µg/well, underscoring their advantage as protein-based delivery systems. In contrast, MSNs induced a more pronounced toxic effect; at 2 µg/well, only 30% cell survival was observed. MSN toxicity is typically linked to oxidative stress and mitochondrial dysfunction, which can trigger cellular apoptosis, as confirmed by flow cytometry (Supplementary Materials, Figure S1) [34,35,36]. Based on these findings, a non-toxic dose of MSNs at 0.5 µg per well was selected. While this concentration is more than an order of magnitude lower than that for ANPs, it is important to note that NP internalization can significantly diverge from the selected doses due to different physicochemical properties like the surface charge. To assess intracellular uptake, NPs were labeled with a fluorescent dye (Cy5.5). The fluorescent signal was then measured after a 6 h incubation of cells with their respective non-toxic doses (10 µg/well for ANPs and 0.5 µg/well for MSNs, with a cell density of 10 × 10^3^ cells per well) (Figure 2b). Analysis of the resulting images revealed comparable intracellular concentrations for both NP types, indicating efficient internalization of silica despite the lower external dose. This equivalency in intracellular NP concentration validates the subsequent assessments, ensuring that any observed lysosomal effects are attributable to the intrinsic physicochemical properties of the nanoparticles rather than differences in their cellular accumulation or impact on cell viability.

### 2.3. Effect on Lysosomes

**Figure 3:**
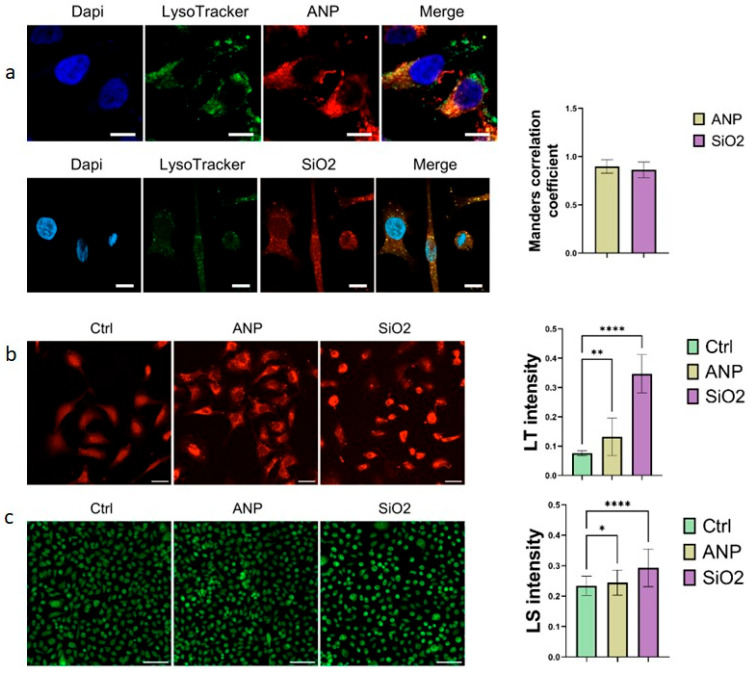
NPs’ effect on lysosomes: (**a**) localization of NPs in lysosomes (red—NPs, green—lysosomes, blue—nuclei; colocalization is marked in yellow in the Merge channel.) after 6 h of treatment and overnight incubation. Scale bars correspond to 10 µm. Histogram presents the respective tM1 Manders’ coefficient values. Confocal images of SKBR3 cells stained with (**b**) Lysotracker Red and (**c**) LysoSensor green after 6 h of NP treatment and 42 h of incubation. Scale bars correspond to 100 µm. For each experiment, 3 biological and 3 technical replicates were performed. Results represent mean ± SD. * = *p* < 0.05, ** = *p* < 0.01, **** = *p* < 0.0001.

The primary route for nanoparticle uptake by cells is endocytosis [37,38], which directs to newly formed lysosomes. Both types of nanoparticles showed strong colocalization with LysoTracker 48 h after NP treatment, with Mander’s correlation coefficients exceeding 0.85 for each. Additionally, both nanoparticle types caused a significant increase in LysoTracker fluorescence intensity, suggesting either an increase in lysosome number or enhanced lysosomal acidity (Figure 3b). However, quantitative measurements of lysosomal pH using LysoSensor revealed no significant changes following NP treatment (Figure 3c). These results indicate that NP exposure primarily affects lysosome quantity rather than acidity. Furthermore, the nanoparticle type, material composition, and physicochemical properties significantly influence the extent and dynamics of this lysosomal response. Notably, MSNs induced a markedly stronger increase in lysosome number—nearly twice that observed with ANPs.

### 2.4. Effect on Catepsins (CtsB and CtsD) Expression and Activity

**Figure 4:**
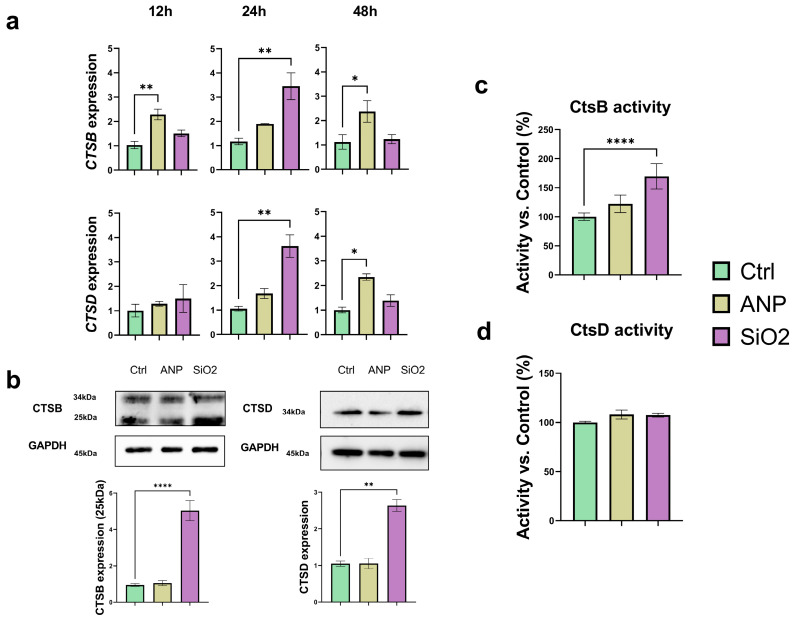
NPs’ effect on CtsB and CtsD expression and activity: (**a**) Changes in CTSB and CTSD gene expression after 12, 24, and 48 h of NP treatment (treatment for 6 h by following washing). (**b**) Protein expression of CtsB and CtsD (normalization with GAPDH) after 6 h NP treatment. Samples were collected after 48 h. (**c**,**d**) Changes in CtsB and CtsD activity after 6 h NP treatment; samples were collected after 48 h. For each experiment, 3 biological and 3 technical replicates were performed. Results represent mean ± SD. * = *p* < 0.05, ** = *p* < 0.01, **** = *p* < 0.0001.

Due to their localization in lysosomes, we investigated whether NPs could affect the expression of lysosomal enzymes, particularly cathepsins. The effects of NPs on CtsB and CtsD were evaluated by measuring their gene expression, protein levels, and enzymatic activity. Changes in gene expression were assessed at multiple time points (Figure 4a). At each time point, NP exposure caused significant alterations in gene expression profiles. For CtsB, both types of NPs increased gene expression more than two-fold at 12 h. ANPs induced a two-fold increase in CtsB expression at all the time points considered, with a slight variation between 24 and 48 h. MSNs, however, exhibited a distinct pattern: CtsB expression peaked with a three-fold increase at 24 h, before returning to baseline levels by 48 h. For CtsD, ANPs induced a time-dependent increase in mRNA expression, with a significant 2.5–fold rise observed at 48 h. MSNs showed a similar pattern to CtsB, with a peak at 24 h and a return to control levels by 48 h. Overall, MSNs had the strongest impact on gene expression for both cathepsins, with increases exceeding 300% (Figure 4a).

Western blot analysis, performed 48 h post-treatment, revealed that for ANPs, the variations in CtsB gene transcription did not result in increased protein translation. In contrast, MSN treatment led to increased protein expression. In this case, we could investigate both immature and mature forms of CtsB, which showed similar trends. For CtsD, only the mature form was detected, and MSN treatment was the only condition that significantly increased its protein expression (Figure 4b). The enzymatic activity of CtsB and CtsD was measured 48 h after NP internalization (Figure 4c,d). Despite the significant increase in CtsD expression induced by MSNs, this did not translate into higher enzymatic activity, and neither NP type significantly affected CtsD activity. In contrast, both NP types influenced CtsB activity, consistent with the gene and protein expression data. Specifically, ANPs generated a slight increase in CtsB activity, while MSN treatment nearly doubled it.

### 2.5. Effect on Cathepsins Localization

**Figure 5:**
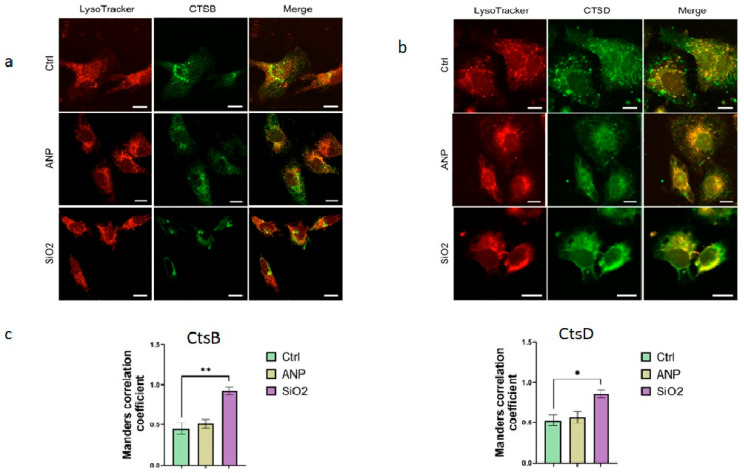
Immunohistochemistry for cathepsin B (**a**) and cathepsin D (**b**) after NP treatment. SKBR3 cells were exposed to NPs for 6 h, followed by an additional 42 h incubation. Cells were stained with LysoTracker (red), fixed, and stained with FITC-labeled antibodies to cathepsins (green); colocalization is marked in yellow in the Merge channel. Scale bars correspond to 10 µm. (**c**) Histograms present the respective Manders’ coefficient values of lysosome and cathepsin colocalization for each experiment; 3 biological and 3 technical replicates were performed. Results represent mean ± SD. * = *p* < 0.05, ** = *p* < 0.01.

Analysis of cathepsin localization revealed no significant changes following nanoparticle exposure (Figure 5). Lysosomes maintained their typical perinuclear distribution, and no evidence of lysosomal membrane permeabilization was observed. A strong colocalization between lysosomes and cathepsins persisted, indicating that lysosomal enzymes were not leaking into the cytoplasm. The higher colocalization coefficients seen with MSNs likely reflect their impact on both lysosome number and cathepsin protein expression levels. Changes in fluorescence intensity within the cathepsin channel further support the Western blot findings, showing a modest increase in protein expression after MSN treatment. For CtsB, the antibodies used detect both the precursor and active forms of the protein, which may dampen the apparent increase in expression since the precursor form is not significantly affected by the nanoparticles.

## 3. Discussion

The interaction of nanocarriers with the cell and their sequestration in the lysosomal compartment is a key determinant affecting the efficacy and safety of drug delivery systems. Lysosomes are central hubs for intracellular degradation and recycling. Following endocytosis, nanocarriers such as albumin and silica nanoparticles are predominantly trafficked to lysosomes, where they accumulate and interact with lysosomal enzymes. In this scenario, it can be assumed that nanocarriers can induce various catabolic pathways and metabolic phenotypes in cancer cells. In this work, two types of nanocarriers were synthesized: albumin and silica nanoparticles, which were characterized by a similar size (~100 nm) and spherical shape. This particle size is optimal for use in drug delivery [39]. Nanoparticle size critically influences biodistribution and cellular uptake: particles < 10 nm undergo rapid renal clearance [40], while those > 200 nm are quickly cleared by the MPS [41]. Optimal cellular internalization often occurs around 50 nm via caveolae-dependent endocytosis, with 50–100 nm particles entering via clathrin-mediated endocytosis, and larger particles (>150 nm) through phagocytosis [42]. Therefore, our similarly sized particles should interact comparably with cancer cells. In contrast, surface charge will lead to distinct behaviors: negatively charged albumin particles versus positively charged silica nanoparticles (Figure 1). A positive charge enhances cellular internalization due to membrane interactions and favors clathrin-mediated endocytosis, whereas a negative charge promotes caveolae-mediated endocytosis [43]. Crucially, a positive charge also facilitates lysosomal escape, vital for non-degradable delivery systems [44].

ANPs offer high delivery efficiency and enhanced safety due to their endogenous origin, which provides inherent biocompatibility, biodegradability, low toxicity, and reduced immunogenicity, resulting in prolonged circulation time and improved cell viability compared with synthetic carriers [45]. Our data confirm high bioavailability, as even 500 µg/well doses induce no significant cytotoxicity, enabling maintenance of therapeutic payload concentrations due to extended half-life. MSNs are widely used delivery systems valued for their tunable physicochemical properties and porous structures, and are FDA-approved as biocompatible carriers with various modifications to improve bioavailability and biodegradability [46]. However, MSNs exhibit significant cytotoxicity at doses above 0.5 µg/well, primarily due to oxidative stress caused by silicon surface groups generating excessive ROS, immune activation through proinflammatory cytokine release, and membrane disruption leading to increased permeability and cell death [47].

Cytotoxicity was evaluated to identify non-lethal doses of nanotherapeutics: while ANPs showed strong biocompatibility, MSNs were effective in reducing cell viability. For this reason, the cells were exposed to different doses of NPs, exceeding an order of magnitude (10 µg/well vs. 0.5 µg/well or 1 vs. 0.05 ng/cell for ANPs and MSNs, respectively). However, comparable nanoparticle internalization occurred (Figure 2b), attributable to MSNs’ positive charges, increasing nanoparticle uptake, as shown by different papers [25]. Quantitative assessments are presented in Figure S2 of Supplementary Materials. All experiments employed a 6 h nanoparticle exposure followed by washing and incubation in fresh medium. While initial nanoparticle binding/internalization occurs rapidly (seconds–minutes), this represents only early uptake [48]. Subsequent intracellular processing—including vesicle formation, compartmental trafficking, and cellular accumulation—requires extended durations to establish fully measurable states, as previously shown [23].

Following endocytic uptake, NPs accumulate within lysosomes—organelles responsible for degradation—promoting increased lysosomal volume and number as cells attempt to process foreign particles. Analysis of colocalization was performed with Manders’ coefficient (Figure 3a) and masking mode (Figure S3 of the Supplementary Materials). The adaptation mechanism to NP-induced stress during early stages may influence lysosomal biogenesis [49]. Most studies report LysoTracker signal alterations under prolonged NP exposure. For instance, Gold NPs reduced LysoTracker signal at 4 h, with recovery by 24 h, suggesting cellular adaptation [50]. A similar effect was shown for particles able to disrupt endosomal vesicles and cytoplasmic delivery [51]. On the other hand, Fe_2_O_3_ NPs increased fluorescence intensity, linked to oxidative stress and heightened lysosomal activity [52]. In this work, both the inorganic MSNs’ and biological ANPs’ effect on the lysosomal compartment and our data confirm that MSNs increase the LysoTracker RED signal (Figure 3b), indicating lysosomal vesicle increase. However, under our experimental conditions, this phenomenon was not accompanied by pH alteration—contrasting with the typical lysosomal dysfunction response to inorganic particles [53]. Gold NPs induced lysosomal alkalization, impairing degradation capacity and causing autophagosome accumulation [54]. Conversely, NPs preserved lysosomal pH (quantified by LysoSensor; Figure 3c); a slight decrease in the pH was observed for both types of NPs and, in particular, for MSNs. Lysosomal functionality critically depends on enzymatic composition, maintaining cellular homeostasis through proteolytic activity mediated by cathepsins. In this study, the focus was on two types of cathepsins. CtsB plays key roles in protein turnover, apoptosis and autophagy [55], and CtsD contributes to autophagy and endocytosis, supporting cellular homeostasis beyond its lysosomal functions [56]. The expression and localization of CtsB and CtsD reflect cellular proteolytic activity and stress responses, making them valuable targets for designing smart drug delivery systems [57]. Both types of particles used in this study are capable of influencing the enzymatic composition of lysosomes. In a previous work, we showed that ANPs can be degraded within lysosomes [58], in turn affecting the expression levels of lysosomal enzymes [23]. The efficiency of degradation and impact on enzyme expression depend on the amount of cross-linker; in this study, we utilized stable nanoparticles exhibiting high degradation efficiency. On the other hand, MSNs, which are resistant to degradation, can initiate a cascade of biological processes that ultimately alter protein levels [59]. These changes have been observed at both the protein and mRNA levels.

A recent study demonstrated that MSNs disrupt autophagy-mediated protein turnover and impair degradation pathways by interfering with cathepsin activity [60]. Analysis of various MSN concentrations revealed distinct effects on the expression of CtsB and CtsD after 24 h of exposure in healthy and cancer cells. In healthy cells, high doses of MSNs resulted in decreased CtsB gene expression, whereas in cancer cells, gene expression levels increased. Interestingly, both cell types showed dose-dependent reductions in CtsB protein expression. No significant alterations in CtsD expression levels were detected following MSN exposure. The authors propose that MSNs may activate CtsB transcription while simultaneously promoting post-transcriptional protein degradation; however, the underlying mechanisms remain unclear.

Our previous research identified a correlation between proteolytic activity and the resistance of ANPs to degradation: ANPs increased CtsB expression and moderately elevated CtsD levels [23]. While existing studies confirm the role of CtsD in ANP degradation, the reverse effect—ANP-mediated regulation of CtsD—has not been reported [61,62]. Notably, prolonged exposure to ANPs in renal podocytes reduces CtsB activity, impairing lysosomal proteolysis and increasing cytokine release. This finding is clinically significant, as CtsB and CtsL are critical for albumin degradation and maintaining podocyte integrity. Reduced enzyme activity leads to albumin accumulation and exacerbates podocyte damage [63].

Both ANPs and MSNs exhibit a similar overall pattern of influence on cathepsins, marked by increased expression of the CtsB and CtsD genes, albeit at varying rates. Notably, MSNs induce more pronounced changes—an unexpected finding given their non-degradable nature. Typically, the impact of MSNs on cathepsins is limited to lysosomal permeabilization and the subsequent release of cathepsins into the cytoplasm, but this phenomenon was not observed in our experimental conditions. In contrast, we expected that ANPs positively regulated cathepsin protein expression, as these enzymes are directly involved in the lysosomal degradation of albumin, the accumulation of which naturally upregulates the gene expression of these enzymes.

Protein expression was evaluated at 48 h, revealing that changes in protein abundance were generally modest. However, MSNs had a stronger effect on CtsB protein levels and activity, with enzymatic activity increasing by more than 50%. In contrast, changes in CtsD expression were not accompanied by a significant increase in enzymatic activity.

It is important to note that introducing any nanocarrier into the bloodstream creates a complex system due to opsonization, leading to the formation of a protein corona. The composition of this corona depends on the nanoparticle material, size, and shape, and it can significantly influence cellular interactions. To eliminate the confounding effects of protein corona formation, control experiments were conducted in serum-free culture medium during the 6 h internalization phase (Figure S4, Supplementary Materials). The results showed that the absence of a protein corona did not alter the observed trends, with MSNs consistently exerting the greatest effect on lysosomal enzymatic composition (Figures S5 and S6, Supplementary Materials). The protein corona composition of the nanoparticles studied in this work may differ significantly: ANPs predominantly adsorb homologous proteins, which reduces nonspecific binding, including interactions with cathepsins. In contrast, MSNs form a more diverse and dynamic protein corona, leading to competitive binding between proteins and enzymes and potentially promoting cathepsin adsorption on the particle surface. Observed differences in the experiments conducted with and without the protein corona suggest its possible role in modulating proteolytic activity; however, the intrinsic material properties of the nanoparticles exert a more dominant influence. These preliminary findings will serve as the foundation for our future studies, focusing on the supramolecular basis of protein corona formation.

In our previous work, similar doses of ANPs induced significant changes in CtsB expression, a result not replicated in the current study [23]. This variation may be due to differences in cell density; the current experiments employed a five-fold lower cell density (0.2 × 10^5^ vs. 1 × 10^5^ cells/cm^2^), mitigating the ability of the cells to interact with the administered particles and consequently their effects on lysosomal biology. However, under these conditions, MSNs still induced significant upregulation of CtsB at both the gene and protein activity levels, while CtsD increased expression was not associated with a substantial increase in their activity.

Due to the non-degradable nature of the nanoparticles, we did not expect them to exert a stronger effect on lysosomal proteases. To elucidate the precise molecular mechanisms underlying silica nanoparticle interactions with cells, more in-depth molecular investigations are required. ANPs, as substrates of cathepsins, undergo enzymatic degradation that triggers localized cathepsin activation but also consumes the enzyme, leading to a regulated expression controlled by lysosomal turnover and feedback from degradation products. In contrast, non-degradable MSNs persist intracellularly, causing sustained cellular stress, which markedly upregulate cathepsin expression. Furthermore, the more diverse and dynamic protein corona formed on MSNs enhances protease recruitment and cathepsin induction. Thus, while ANPs modulate cathepsin expression primarily through substrate degradation, SiNPs elicit stronger, stress-mediated cathepsin upregulation. A plausible molecular mechanism underlying this stress response involves MSNs inducing the release of mature interleukin-1β (IL-1β) [64], which activates the NF-κB signaling pathway. Importantly, the promoter region of the CTSB gene contains an NF-κB binding site, positioning NF-κB as a positive regulator of CTSB expression [65] and establishing a feedback loop that amplifies cathepsin B expression and activity.

The intracellular localization of cathepsins was also examined, since a common consequence of nanoparticle-induced lysosomal membrane permeabilization is an increased presence of cathepsins in the cytoplasm. Analysis of colocalization was performed with Manders’ coefficient (Figure 5) and masking mode (Figure S7 of the Supplementary Materials). However, in our experiments, no such effect was observed: both cathepsin types exhibited strong colocalization with lysosomes confirming the mild levels of our treatment. On the other hand, both nanoparticle types—particularly silica nanoparticles—induced lysosomal rearrangement characterized by pronounced perinuclear clustering. This lysosomal reorganization, including enhanced perinuclear lysosome clustering in response to nanoparticle treatment, has been reported [66] via nuclear translocation of TFEB, that is associated with both lysosomal biogenesis and repositioning. This process often results in lysosomes accumulating in the perinuclear region as part of the cellular adaptive response to lysosomal stress. Such lysosomal redistribution represents a hallmark of lysosomal adaptation aimed at preserving organelle function under nanoparticle exposure and is linked to modifications in lysosomal ion channel activity and pH regulation [67].

## 4. Materials and Methods

### 4.1. Chemicals and Reagents

The human breast adenocarcinoma cell line (SKBR3) was purchased from the American Type Culture Collection. Cells were cultured in RPMI medium (Paneko, Moscow, Russia) supplemented with 10% fetal bovine serum and 1% penicillin–streptomycin antibiotic mixture (Gibco, Waltham, MA, USA) at 5% CO_2_ and 37 °C in a humidified atmosphere. The cells were checked using MycoReport (Evrogen, Moscow, Russia) and were free of contamination. Bovine serum albumin and ethanol were purchased from Sigma-Aldrich (Saint Louis, MO, USA), while glutaraldehyde was purchased from PanReac AppliChem (Barselona, Spain). Tetraethyl orthosilicate, cetriltrimethylammonium bromide, triethanolamine, and 3-aminopropyltriethoxysilane were purchased from Sigma-Aldrich (Saint Louis, MO, USA). Cy5.5-NHS dye for NP labeling was purchased from Lumiprobe (Moscow, Russia). All reagents required for Western blot analysis were obtained from Bio-rad (Hercules, CA, USA), while the antibodies were purchased from Abcam (Cambridge, UK). All the reagents necessary to perform PCR analysis were obtained from Evrogen (Moscow, Russia). LysoTracker Red, LysoTracker Green DND, and Lysosensor Green were obtained from Invitrogen (Waltham, MA, USA).

### 4.2. Nanoparticles Synthesis

Albumin nanoparticles (ANPs) were synthesized via a desolvation process, followed by cross-linking, in accordance with a previously established protocol. At a 4:1 ratio, 95% ethanol was added dropwise to a water solution of BSA (20 mg/mL). At this stage, protein denaturation and nanoparticle formation occur. After that, 0.1% glutaraldehyde in an alcohol solution was added to the solution as a cross-linking agent to stabilize the particles and transfer them to water; the ratio of the additive to the mixture was 1 to 10. This concentration of the cross-linking agent is the minimum for the formation of colloidally stable particles in aqueous solutions. The solution was stirred for 24 h at room temperature. After that, the solution was washed from free ethanol precursors by centrifugation 3 times and stored in an aqueous solution with a concentration of 10 mg/mL.

Synthesis of mesoporous silica nanoparticles (MSNs) was carried out using sol–gel technology, which allows control of the formation of particles with certain physicochemical properties. The synthesis took place in two stages. In the first stage, a mixture containing TEA and CTAB was prepared with concentrations of 1.75 g/L and 8.3 g/L, respectively. The mixture was stirred for 30 min at 80 °C. In the second stage, TPOS and APTES were added to the resulting mixture to a final concentration of 11.15 g/L and 1.25 g/L. The resulting solution was stirred at 80 °C for 2 h, then allowed to cool to room temperature. Free precursors were removed, and the sample was concentrated by centrifugation following the addition of ethanol and acetic acid; this process was repeated twice.

### 4.3. Nanoparticles Characterization

ζ potential and particle size distribution were measured using a Zetasizer Nano ZS automated analyzer (Malvern Instruments, Malvern, UK) at room temperature (25 °C). Samples were diluted multiple times to prevent NP aggregation and interactions until consistent and stable data were achieved. Scattering measurements were performed at a detection angle of 173 °C. The average size was calculated using the Stokes–Einstein equation based on the velocity of Brownian motion estimated from fluctuations in the intensity of scattered light. The recalculation of the average size by the number of particles is carried out on the basis of the Mie theory. Zeta potential measurements were obtained using the Smoluchowski model to ensure accurate assessment of particle surface charge. The morphology and average size of NPs were characterized by scanning electron microscopy (SEM). SEM analysis was performed using a Crossbeam 550 instrument (Carl Zeiss, Jena, Germany).

### 4.4. Nanoparticles Toxicity

MTT assay was used to evaluate nanoparticle impact on cell viability. The cells were seeded in 96-well microplates (Costar, Corning Inc., Corning, NY, USA) at a density of 10 × 10^3^. Twenty-four hours after cell attachment, plates were washed with PBS, and the cells were exposed to varying NP concentrations for 72 h (with a treatment volume of 100 µL). For each control and test concentration, six replicate wells were utilized. The tetrazolium salt MTT [3-(4,5-dimethylthiazol-2-yl)-2,5-diphenyltetrazolium bromide] was prepared at a concentration of 5 mg/mL in PBS and added to SKBR3 cells (100 μL per mL of DMEM lacking serum and phenol red) following the protocol established by Mosmann. Following a 2 h incubation at 37 °C, the plates were washed with PBS, and DMSO was added to each well and mixed to dissolve the dark-blue formazan crystals. After allowing the plates to stand at room temperature for several minutes, absorbance was measured at 570 nm using a CLARIOstar^®^ Plus reader (BMG Labtech, Ortenberg, Germany).

### 4.5. Nanopartciles Localization and Internalization

The analysis of the internalization of nanoparticles into cells was performed using a confocal microscope LSM 980 Airyscan (Carl Zeiss, Jena, Germany). For this purpose, nanoparticles were labeled using the dye Cy5.5-NHS (Lumiprobe, Moscow, Russia). To prepare the labeling reagent, the dye powder was dissolved in DMSO to achieve a concentration of 2.5 mg/mL. The requisite quantity of dye—specifically, 2.5 μg of dye per 10 mg of ANPs or 7 mg of MSNs—was introduced into the NP suspension to achieve equivalent fluorescence quantum yield, maintaining the pH of the solution at 8.5 to facilitate optimal conjugation. The mixture was continuously stirred in a dark environment at room temperature for a period of 24 h. Subsequent to the incubation, unbound dye was removed through centrifugation.

To assess internalization, 30 × 10^4^ cells were plated on a cover glass in a 3 cm Petri dish. Then, 24 h post cell attachment, the plates were rinsed with PBS, and the cells were exposed to varying concentrations of NPs for 6 h, followed by washing with PBS to eliminate non-internalized particles. After incubation for 42 h at 37 °C plates were washed with PBS, fixed with 2% solution of paraformaldehyde in PBS for 15 min at room temperature, and washed with PBS. To assess intracellular localization, a similar approach was used: one day after treatment with labeled NPs, the cells were incubated with LysoTracker Green DND for 30 min at 37 °C; then, washed twice with PBS and fixed with paraformaldehyde, after washing, cells were stained with DAPI nuclear dye, incubated for 5 min, and then washed again with PBS. Confocal microscopy preparations were prepared using Antifade Solution and fixed in the dark at room temperature overnight.

### 4.6. Analysis of Catepsins’ Expression and Activity

For all experiments, 30 × 10^4^ SKBR-3 cells were plated in a 6 cm Petri dish. Following 6 h of particle treatment, the culture medium containing NPs was discarded. After incubation at 37 °C for various periods of time for gene expression analysis and for 48 h for cathepsins expression and activity analysis.

#### 4.6.1. RNA Isolation Real-Time Polymerase Chain Reaction (RT-qPCR)

Total RNA was isolated from cells using the PureLink™ RNA Kit (Invitrogen, Waltham, MA, USA) according to the manufacturer’s instructions. Subsequently, complementary DNA (cDNA) was synthesized from mRNA utilizing a cDNA MMLV kit (Evrogen, Moscow, Russia). For the reverse transcription reaction, one microgram of total RNA with an optical density (OD260/OD280) between 1.7 and 2.0, measured using a NanoDrop One (ThermoFisher, Waltham, MA, USA), was employed. Expression of the human genes was quantified by RT-qPCR using the cDNAs as templates in reactions containing the DNA-specific dye 5X qPCRmix-HS (Evrogen, Moscow, Russia) and specific oligonucleotide primers (CTSB: F-5′-TTCTTGCGACTCTTGGGACTTC-3′, R-5′-TGACGAGGATGACAGGGAACTA-3′; CTSD: F-5′- GGACTACACGCTCAAGGTG-3′, R-5′- GTTGTCACGGTCAAACACAG-3′; GAPDH: F-5′-CTTCGCTCTCTGCTCCTCCTGTTCG-3′, R-5′-ACCAGGCGCCCAATACGACCAAAT-3′). PCR reactions were conducted in triplicate under the following conditions: an initial denaturation at 95 °C for 30 s, followed by 40 cycles of 95 °C for 5 s, 60 °C for 15 s, and 72 °C for 10 s, using the StepOne Real-Time PCR System (Applied Biosystems, Waltham, MA, USA). Gene expression levels were evaluated using the ddCT method.

#### 4.6.2. Western Blotting

Following transfection, the cells were washed twice with ice-cold PBS, collected, and resuspended in a lysis buffer containing 50 mM Tris-HCl (pH 8.0), 100 mM NaCl, 0.5% NP-40, 1% Triton X-100, and 1× protease inhibitor cocktail (ThermoFisher, Waltham, MA, USA). Protein concentrations in the cell lysates were determined, and 50 μg of each sample was separated on a 12% Tris-glycine gel before being transferred to PVDF membranes (Bio-Rad, Hercules, CA, USA). Expression of the CtsB and CtsD genes was detected using specific primary antibodies: rabbit polyclonal antibodies against CTSB and CtsD (Cloud-Clone Corp., Wuhan, China). After thorough washing, membranes were incubated with goat anti-rabbit IgG (HRP) secondary antibodies (Abcam, UK, Cambridge) in 5% non-fat milk prepared in PBST. Signals from the reactive bands were visualized using enhanced chemiluminescence detection (Bio-Rad, Hercules, CA, USA). As a loading control, membranes were also probed with primary rabbit polyclonal antibodies against GAPDH (Cloud-Clone Corp., Wuhan, Hubei, China) followed by goat anti-rabbit IgG (HRP) secondary antibodies (Abcam, UK, Cambridge).

#### 4.6.3. Cathepsins Activity Assay

Following 48 h after addition of NPs, cells were lysed and activity was assessed using Fluorometric Cathepsin B (ab65300) and Cathepsin D (ab65302) activity kits (Abcam, Cambridgeshire, UK). Cell lysis and sample preparation were performed according to the manufacturer’s protocol. Fluorescence was measured on a CLARIOstar^®^ Plus reader (BMG labtech, Ortenberg, Germany).

### 4.7. Immunostaining of Cathepsins

As in previous experiments, SKBR3 cells were incubated with nanoparticles for 6 h, washed with PBS, and then incubated for an additional 42 h. Initially, lysosomes were stained using LysoTracker RED as described earlier. The cells were subsequently washed with PBS, fixed with 1% PFA in PBS for 15 min, and permeabilized with 0.25% Triton^®^ X-100 in PBS for 10 min. Following the blocking of nonspecific binding sites with 2% BSA in PBS, cells were incubated for 4 h with primary antibodies: rabbit polyclonal anti-CTSB (Cloud-Clone Corp., Wuhan, China) and anti-CTSD (Affinity Biosciences, Cincinnati, OH, USA). After washing with PBS, cells were incubated for 1 h at room temperature with fluorophore-conjugated secondary antibodies. Detection of CTSB and CTSD was performed using FITC-labeled secondary antibodies (Cloud-Clone Corp., Wuhan, China). Nuclei were counterstained with DAPI (Bio-Rad, Hercules, CA, USA). Confocal fluorescence images were captured using an inverted scanning confocal microscope (LSM 980 AiryScan on Axio Observer 7, Carl Zeiss Microscopy GmbH, Jena, Germany) equipped with a motorized piezo stage and 63× oil immersion objectives (Carl Zeiss Microscopy GmbH, Jena, Germany). Fluorescence intensity quantification on 20× images was conducted using the CellProfiler 4.2.4 software, while post-acquisition processing of 63× images was performed in Fiji, utilizing the e2fig plugin.

### 4.8. Statistical Analysis

Statistical analysis was performed using GraphPad Prism 9.5 for Windows (GraphPad Software, San Diego, CA, USA). For real-time PCR, three biological replicates were conducted, each containing three technical replicates per sample. Western blot analysis was performed three times using distinct biological replicates. Confocal microscopy data were collected from three biological replicates, capturing images from multiple areas of each preparation. The data were analyzed using the ANOVA method. Results are presented as mean ± SD for normally distributed data. Statistical significance was assessed using One-Way ANOVA followed by Dunnett’s post hoc test, with *p*-values < 0.05 considered significant. Statistical significance is denoted as *p < 0.05, **p < 0.01, and ***p < 0.001.

## 5. Conclusions

Both albumin and silica nanoparticles exerted significant effects on lysosomes, irrespective of their material composition, with the non-degradable silica nanoparticles unexpectedly producing more pronounced effects at every stage. The enzymatic composition of lysosomes serves as a dynamic indicator of cellular state, influenced by numerous factors beyond nanoparticle exposure. Understanding these interactions is crucial for the rational design of nanocarriers, as lysosomal dysfunction or modulation can significantly impact drug bioavailability, targeting specificity, and cytotoxicity profiles. This study highlights how albumin and silica nanocarriers differentially regulate the expression and localization of cathepsins B (CtsB) and D (CtsD), providing valuable insights for optimizing drug delivery systems to enhance therapeutic efficacy while minimizing adverse effects.

Importantly, these findings underscore a key principle: nanocarriers not only deliver drugs but actively influence cellular metabolism, which can substantially affect therapeutic outcomes. Future research should focus on deepening our understanding of nanoparticle–cell interactions to strike a balance between effective delivery and minimizing off-target effects. This study establishes a foundation for rationally engineering nanomedicines that leverage lysosomal pathways without compromising cellular viability, paving the way for safer and more effective drug delivery systems.

## Data Availability

The original contributions presented in this study are included in the article. Further inquiries can be directed to the corresponding authors.

## Supplementary Materials

The following supporting information can be downloaded at: https://www.mdpi.com/article/10.3390/ijms26178291/s1.
