# Supplementary material for: Inorganic Silica Nanoparticles Increase Lysosomal Biology and Protease Activity
Source: Int J Mol Sci. 2025 Aug 26;26(17):8291. doi: 10.3390/ijms26178291 (PMC12428692; doi:10.3390/ijms26178291)
Supplement: Supplementary file 1 [file ijms-26-08291-s001.zip › ijms-3783311-supplementary.pdf]

Supplementary Information

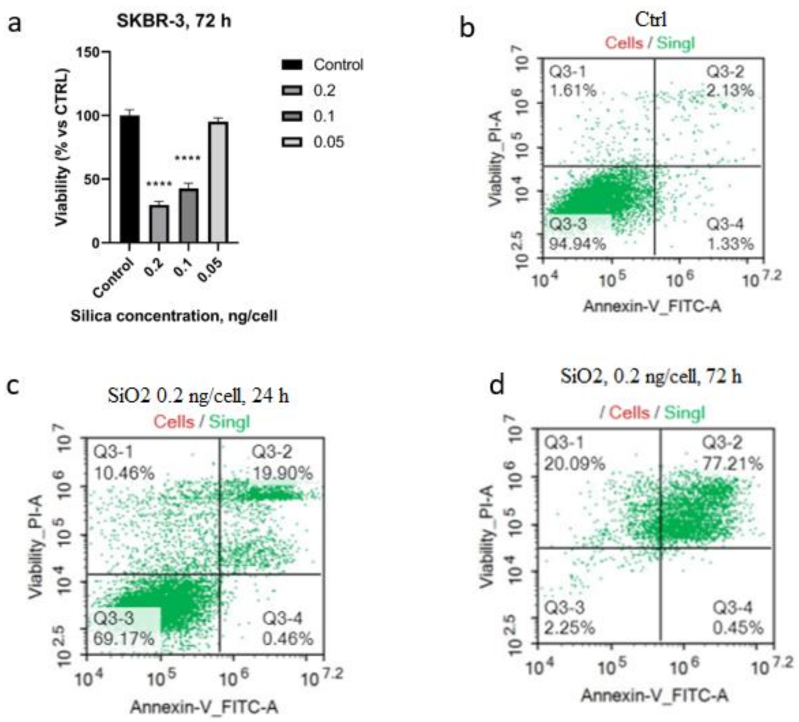

Figure S1. Toxicity of SiNPs: (a) Viability of SKBR3 cells after 72 hours treatment with different doses of SiNPs. FACS with Annexin V-FITC/PI Kit: (b) control; (c) 0,2 ng/cell, 24 hours of treatment; (d) 0,2 ng/cell, 72 hours of treatment.

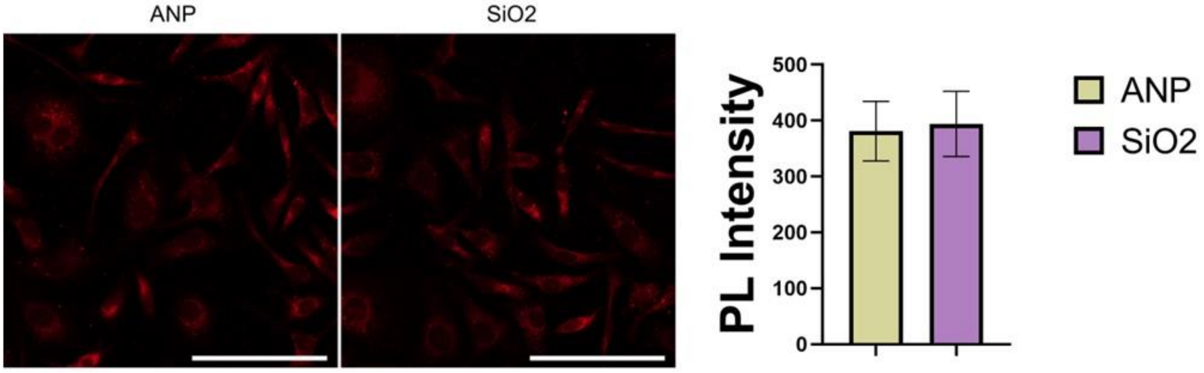

Figure S2. Fluorescence intensity of ANPs and MSNs in SKBR3 cells. Representative fluorescence channel images of nanoparticles in cells with 20× magnification. Scale bars correspond to 100 μm. The nanoparticle fluorescence intensity graph was constructed based on the average fluorescence per cell. (Supplementary to Figure 2b)

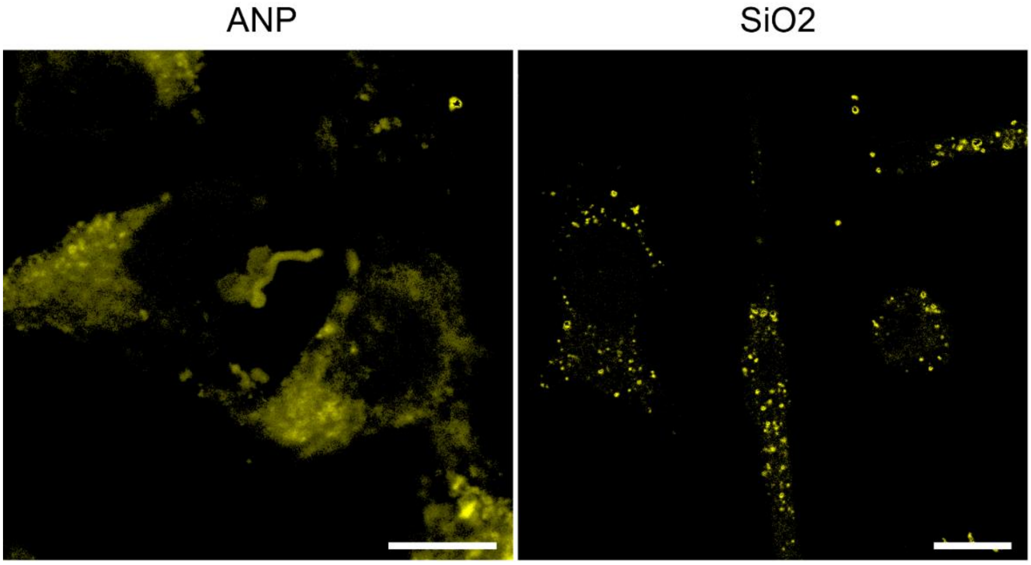

Figure S3. Fluorescence colocalization analysis of lysosomes with ANPs or MSNs in SKBR3 cells was performed using masking in ZEN Blue software. Scale bars correspond to 10 μm.

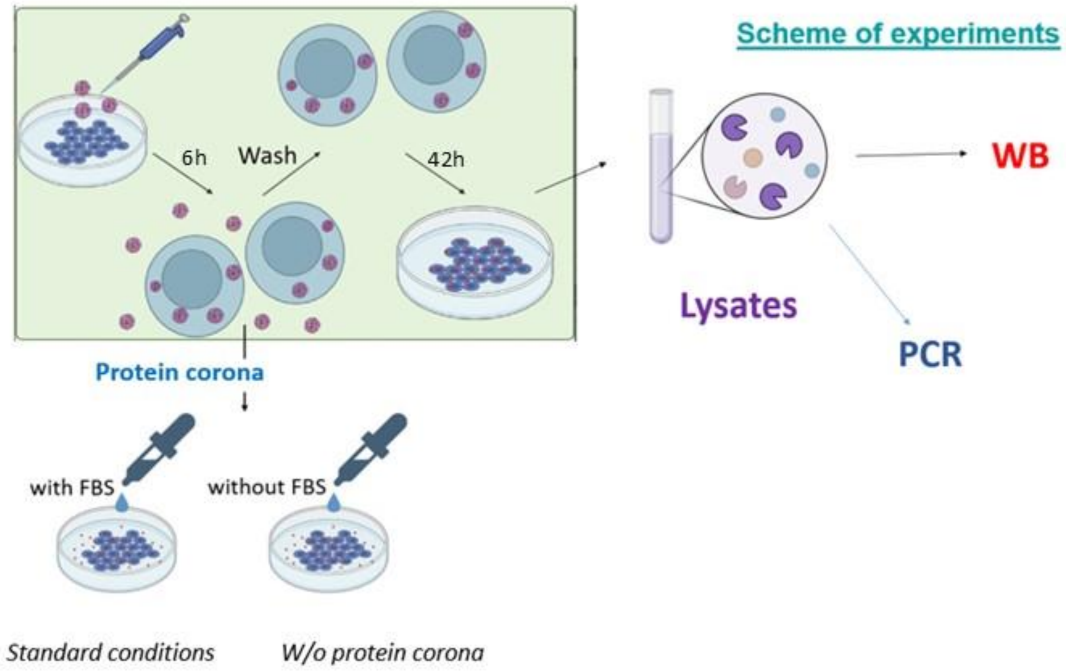

Figure S4. Scheme of the experiment to clarify the influence of the protein corona on NPs surface on the observed effect of NPs on lysosomes: at the stage of cells incubation with NPs (6 h), a medium with or without the addition of FBS was used.

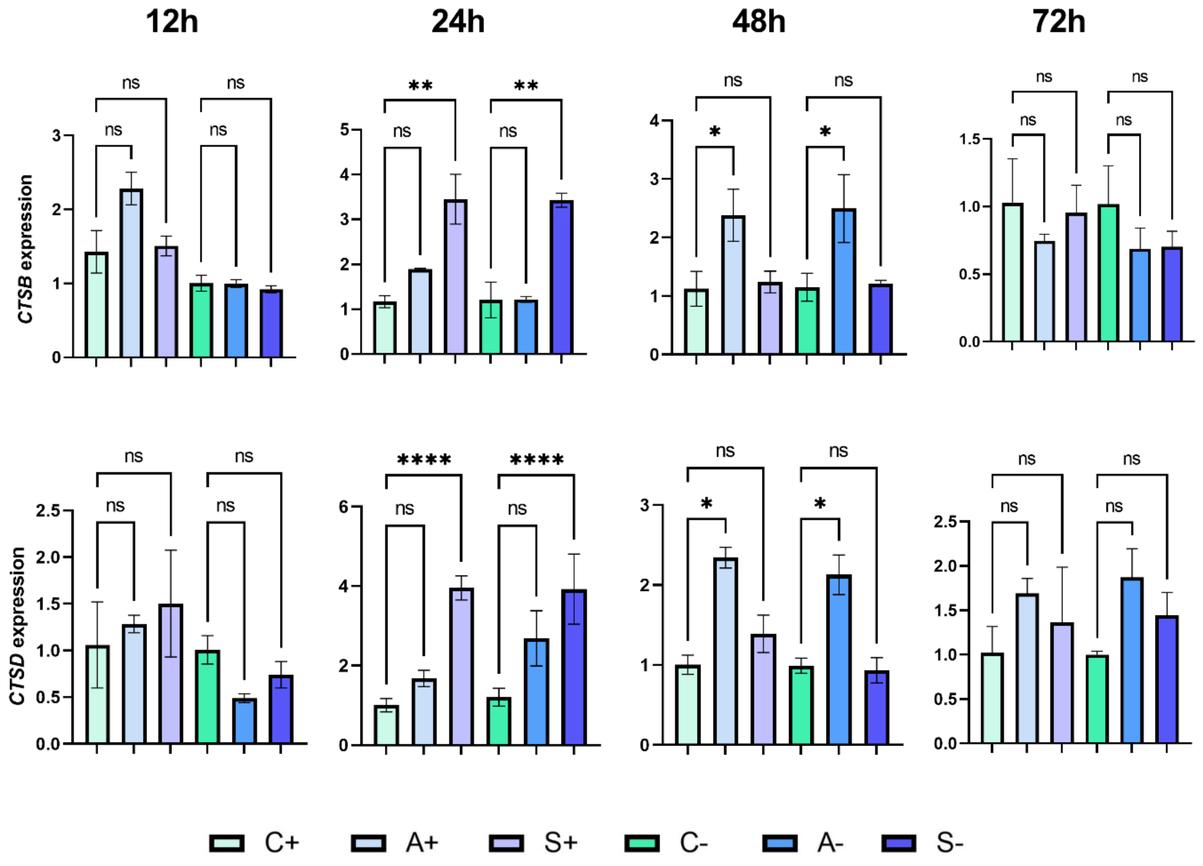

Figure S5. Changes in *CTSB* and *CTSD* gene expression after 12, 24 and 48 h of NPs treatment (treatment for 6 h by following washing); +- protein corona (standard procedure for co-incubation of NPs with cells) – no protein corona (FBS was excluded from the culture medium during incubation); C-control, A-ANPs, S-SiNPs

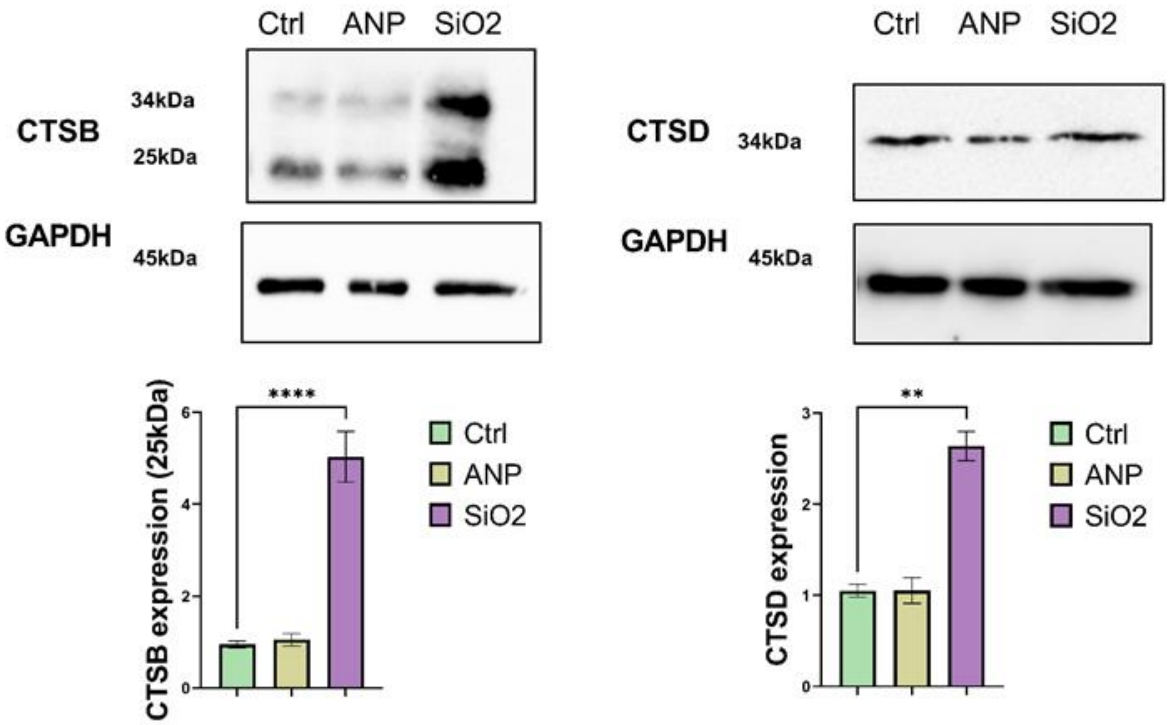

Figure S6. Effect of ANPs and SiNPs without protein corona (FBS was excluded from the culture medium during incubation) on CtsB and CtsD expression

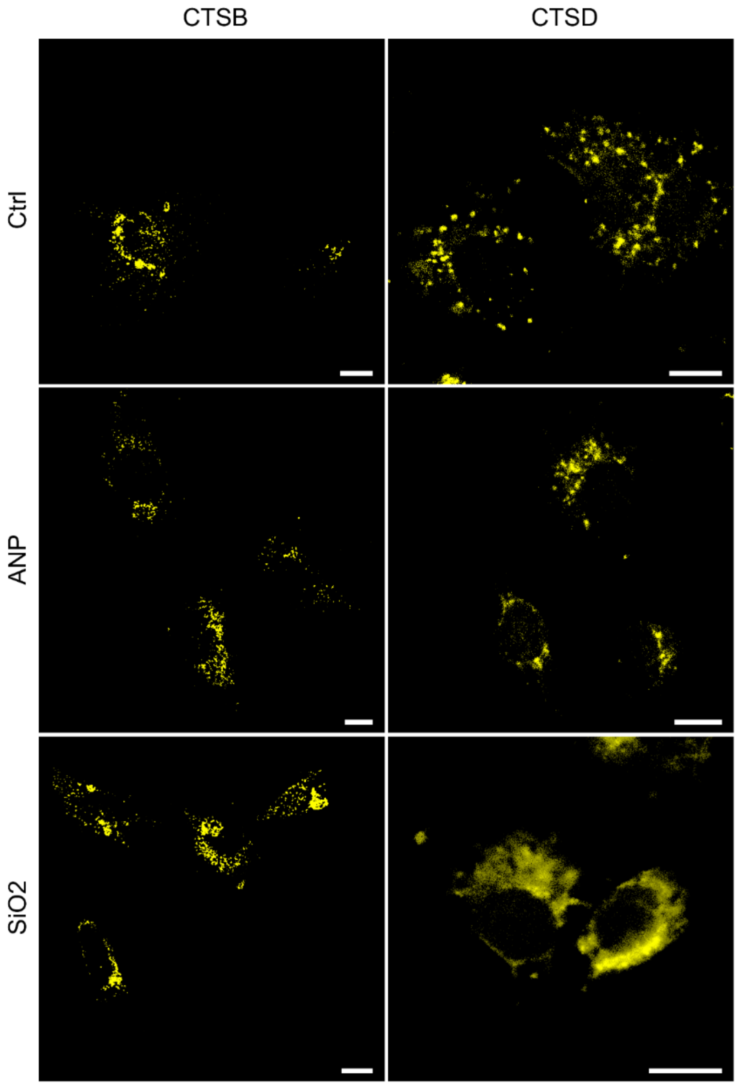

Figure S7. Fluorescence colocalization analysis of lysosomes with CTSB or CTSD in SKBR3 cells was performed using masking in ZEN Blue software. Scale bars correspond to 10 μm.
